# Identification of key gene modules and hub genes of human mantle cell lymphoma by coexpression network analysis

**DOI:** 10.7717/peerj.8843

**Published:** 2020-03-20

**Authors:** Dongmei Guo, Hongchun Wang, Li Sun, Shuang Liu, Shujing Du, Wenjing Qiao, Weiyan Wang, Gang Hou, Kaigang Zhang, Chunpu Li, Qingliang Teng

**Affiliations:** 1 Department of Hematology, Taian City Central Hospital, Taian, Shandong, China; 2 Department of Clinical Laboratory, Qilu Hospital of Shandong University, Jinan, Shandong, China; 3 Department of Occupational Disease, Taian City Central Hospital Branch, Taian, Shandong, China; 4 Department of Pathology, Taian City Central Hospital, Taian, Shandong, China; 5 Department of Orthopedics, Taian City Central Hospital, Taian, Shandong, China; 6 Department of Orthopedics, Tianjin Medical University Cancer Institute and Hospital, Tianjin, China

**Keywords:** Mantle cell lymphoma, Coexpression network analysis, Modules, Hub genes, Survival

## Abstract

**Purpose:**

Mantle cell lymphoma (MCL) is a rare and aggressive subtype of non-Hodgkin lymphoma that is incurable with standard therapies. The use of gene expression analysis has been of interest, recently, to detect biomarkers for cancer. There is a great need for systemic coexpression network analysis of MCL and this study aims to establish a gene coexpression network to forecast key genes related to the pathogenesis and prognosis of MCL.

**Methods:**

The microarray dataset GSE93291 was downloaded from the Gene Expression Omnibus database. We systematically identified coexpression modules using the weighted gene coexpression network analysis method (WGCNA). Gene Ontology (GO) and Kyoto Encyclopedia of Genes and Genomes (KEGG) functional enrichment analysis were performed on the modules deemed important. The protein–protein interaction networks were constructed and visualized using Cytoscape software on the basis of the STRING website; the hub genes in the top weighted network were identified. Survival data were analyzed using the Kaplan–Meier method and were compared using the log-rank test.

**Results:**

Seven coexpression modules consisting of different genes were applied to 5,000 genes in the 121 human MCL samples using WGCNA software. GO and KEGG enrichment analysis identified the blue module as one of the most important modules; the most critical pathways identified were the ribosome, oxidative phosphorylation and proteasome pathways. The hub genes in the top weighted network were regarded as real hub genes (IL2RB, CD3D, RPL26L1, POLR2K, KIF11, CDC20, CCNB1, CCNA2, PUF60, SNRNP70, AKT1 and PRPF40A). Survival analysis revealed that seven genes (KIF11, CDC20, CCNB1, CCNA2, PRPF40A, CD3D and PUF60) were associated with overall survival time (*p* < 0.05).

**Conclusions:**

The blue module may play a vital role in the pathogenesis of MCL. Five real hub genes (KIF11, CDC20, CCNB1, CCNA2 and PUF60) were identified as potential prognostic biomarkers as well as therapeutic targets with clinical utility for MCL.

## Introduction

Mantle cell lymphoma (MCL) is a rare, aggressive malignancy with a low survival rate comprising approximately 6% of non-Hodgkin lymphoma (NHL) cases. MCL patients are typically male with a median age over 60 (Vose, 2017). MCL is largely incurable, although traditional chemotherapy can induce a high rate of remission in previously untreated patients. However, relapse is common within a few years. An intense first-line treatment can improve a patient’s progression-free survival, however, there is still no curative regimen. Therefore, additional insights into the pathology and genetic etiology of MCL may offer new treatment solutions for MCL.

Genetic variations are reportedly related to the occurrence of MCL. MCL is characterized by the presence of *t*(11;14)(q13;q32), which is closely correlated with cyclin D1 overexpression (Ladha et al., 2019). The activation of cell survival pathways and alterations in the DNA damage response contribute to the constitutive dysregulation of the cell cycle and are incorporated to promote the pathogenesis of MCL. There are two different molecular subtypes of MCL, namely the classical MCL and the leukemic nonnodal subtype (Quintanilla-Martinez, 2017). However, the molecular biomarkers of MCL remain unclear. The molecular biomarkers for MCL must be identified to provide targets with which to identify its pathogenesis and to develop personalized treatment strategies. Bomben et al. (2018) have reported that six representative genes (AKT3, BCL2, BTK, CD79B, PIK3CD and SYK) through the analysis of a prediction model were correlated with a poor clinical response in MCL. Ferrero et al. (2019) confirmed mutations of KMT2D and disruption of TP53 had a significantly increased risk of progression and death by target resequencing and DNA profiling in MCL samples. The pathogenesis of MCL is a result of complex molecular mechanisms involving genetic factors, which have not yet been elucidated.

The majority of studies to date have focused on the differential expression of genes associated with MCL but have ignored their high degree of interconnectivity. Expression profiling analysis based on microarrays contributes to measuring gene expression at the genome-wide level. The weighted gene coexpression network analysis (WGCNA) is used to analyze a biological system and can detect an array of genes with similar expression levels as well as their relevant biological functions in diverse physical processes. These analyses can be defined as one gene module. WGCNA can identify the gene modules related to clinical diagnoses and has been used comprehensively in cancer-related research, including for cancers of gastric region and soft tissue (Gong et al., 2019; Zhu et al., 2019).

We constructed a coexpression network from a dataset consisting of 20,822 genes of 123 human MCL samples. Enrichment analysis of Gene Ontology (GO) and Kyoto Encyclopedia of Genes and Genomes (KEGG) were conducted to analyze the gene functions in the six constructed coexpression modules. The protein–protein interaction (PPI) networks were constructed and visualized using the Cytoscape software and the hub genes in the top weighted network were identified. Survival analysis revealed that seven genes (KIF11, CDC20, CCNB1, CCNA2, PRPF40A, CD3D and PUF60) were associated with overall survival time. The informative genes identified in this research may contribute to the future clinical treatment of MCL.

## Materials and Methods

### Microarray data analysis

Analysis was performed on the raw gene expressions of the MCL datasets and the corresponding clinical follow-up obtained from the GEO data repository (http://www.ncbi.nlm.nih.gov/geo). GSE93291, a much larger and newer microarray dataset of MCL, included a total of 123 samples. Another dataset of GSE132929 was downloaded to verify the stability of the real hub genes. This dataset included 43 MCL samples. GPL570 (Affymetrix Human Genome U133 Plus 2.0 Array) was selected for the microarray. Gene IDs were mapped to the microarray probes using the annotated information offered by the record. Probes corresponding to more than one gene were excluded from the dataset. The average expression values of the genes was obtained using measurements from a number of probes. A suitable threshold value was selected based on the number of probes with different thresholds of expression. The WGCNA algorithm (Langfelder & Horvath, 2008) was applied to build the coexpression network. Samples cluster analysis was performed using the hclust tool (R package, https://www.rdocumentation.org/packages/stats/versions/3.6.1/topics/hclust) with a threshold value of 67 in GSE93291.

### Coexpression modules construction

The power value was screened out during the construction of the modules using the WGCNA package in R (https://cran.r-project.org/web/packages/WGCNA/). The mean connectivity and scale independence of network modules were analyzed using the gradient test under different power values, which ranged from 1 to 20. The soft threshold power of 8 was selected according to the scale-free topology criterion. The WGCNA algorithm further identified coexpression modules under these conditions. The minimum size of the gene group was set at 50 to ensure the reliability of the results for this module.

### Interaction analysis of coexpression modules

The interactive relationship among the coexpression modules was studied using the WGCNA algorithm. The WGCNA R software package can be used to determine network construction, the calculation of topological properties, gene selection, module detection, differential network analysis, and network statistics. We chose a height cut of 0.25 (red line), which corresponds with a correlation of 0.75, to merge similar modules. A heat map was drawn to display the intensity of the interaction among the modules.

### Analysis of functional and pathway enrichment

Functional enrichment analysis was carried out in coexpression modules. The genetic information of the respective modules was mapped to the associated GO terms and KEGG pathways using the DAVID tool (version 6.8; http://david.abcc.ncifcrf.gov/) (Huang, Sherman & Lempicki, 2009). The top five records with *p*-value < 0.05 were retained for analysis.

### Hub gene analysis and identification

The genes in the coexpression modules were uploaded to the Search Tool for the Retrieval of Interacting Genes/Proteins (STRING) online database (version 11.0) to evaluate PPI information and construct a functional protein association network (Szklarczyk et al., 2017). Interactions with combined scores above 0.9 were considered significant. The PPI networks were constructed and visualized using Cytoscape software (version 3.7.1; http://www.cytoscape.org/) (Su et al., 2014). PPI hub genes were detected using the cytoHubba plugin with the degree of connectivity set to the top 5%. The weighted networks of the top genes were also ranked by weighted degree in each coexpression module and were visualized using Cytoscape software. The PPI hub genes in the top weighted network were considered to be real hub genes. The validation was performed in the GSE132929 dataset. We used the same approach to detect the real hub genes.

### Survival analysis

Survival analysis was performed for the real hub genes using the R package of survival (https://CRAN.R-project.org/package=survival). The samples were categorized into high and low groups according to the median expression value of each real hub gene. Kaplan–Meier analysis and the log-rank test were used to analyze the correlation between the expression of the real hub gene and its corresponding prognostic information. Significance was considered to be *p* < 0.05.

## Results

### MCL dataset pre-processing

**Figure 1:**
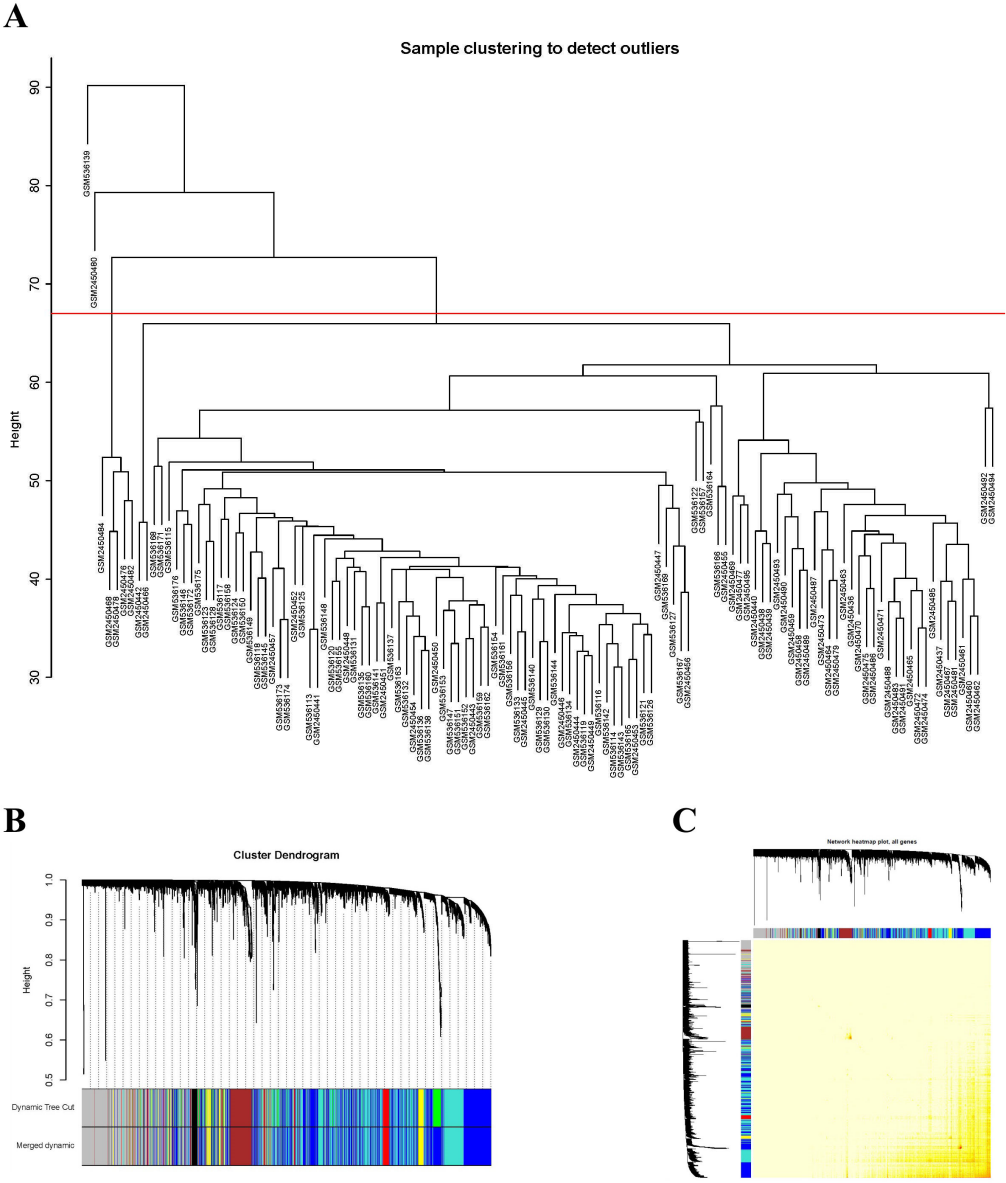
Sample clustering to detect outliers and construction of coexpression modules. (A) Hierarchical clustering of the top 5,000 genes with the highest average expression values of MCL samples in the clustering analysis. There were two outlier samples in the total 123 samples, that are GSM536139 and GSM2450480, when the threshold value was determined as 67 (red line). (B) The constructed coexpression modules of MCL genes by WGCNA software. (C) Interaction analysis between gene coexpression modules. The heatmap showed the Topological Overlap Matrix (TOM) among genes in the analysis. Different colors on the *x*-axis and *y*-axis represented different modules. The yellow brightness of the middle part represented the strength of connections between modules.

A total of 20,822 gene expression values were derived from the raw file. A total of 5,000 genes with the greatest average expression values were selected for cluster analysis (Fig. 1A). A total of 121 samples remained for subsequent analysis after two outlier samples were removed (GSM536139 and GSM2450480).

### Identification of coexpression modules of MCL genes

The expression values of 5,000 genes in the 121 MCL samples were analyzed to identify the modules of highly correlated genes. The soft threshold power was set at 8 (scale-free *R*^2^ = 0.85) to guarantee a scale-free network (Fig. S1). A total of seven modules, including turquoise (1,571 genes), gray (883 genes), blue (1,606 genes), brown (381 genes), yellow (241 genes), red (195 genes) and black (123 genes) were identified (Fig. 1B). The genes in gray were not included in any module, so no further analysis was conducted for these genes.

### Correlation analysis of coexpression modules

**Figure 2:**
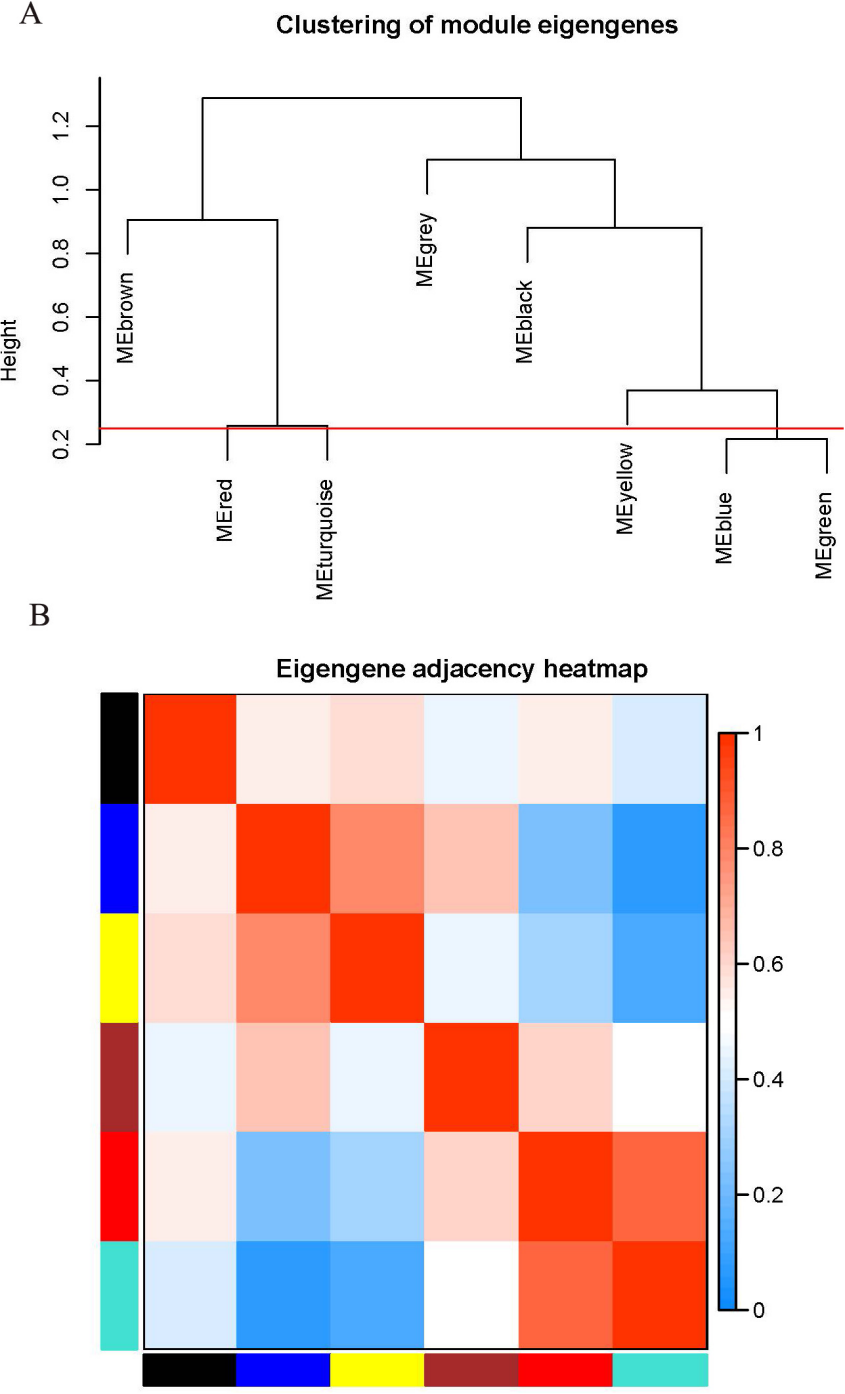
Connectivity analysis between different modules. (A) Hierarchical cluster analysis of the genes in different modules; (B) connectivity level analysis of the genes in different modules. Within the heatmap, red represents a positive correlation and blue represents a negative correlation. Squares of red color along the diagonal are the meta-modules.

The WGCNA package analyzed the interactive relationships underlying the six coexpression modules (Fig. 1C). Gene expression among the six identified modules was relatively independent as illustrated by the topological overlap matrix (TOM) plot of 5,000 genes, suggesting that each module was independently validated. The connectivity degree of eigengenes was analyzed to further quantify the similarity of coexpression. The six modules yielded two main clusters, with two sets of three modules each (brown, red and turquoise modules, and black, blue and yellow modules), followed by cluster analysis (Fig. 2A). The blue and yellow modules, and red and turquoise modules were found to have higher adjacency values based on the heatmap plot of the adjacencies (Fig. 2B).

### Functional and pathway enrichment analysis

**Figure 3:**
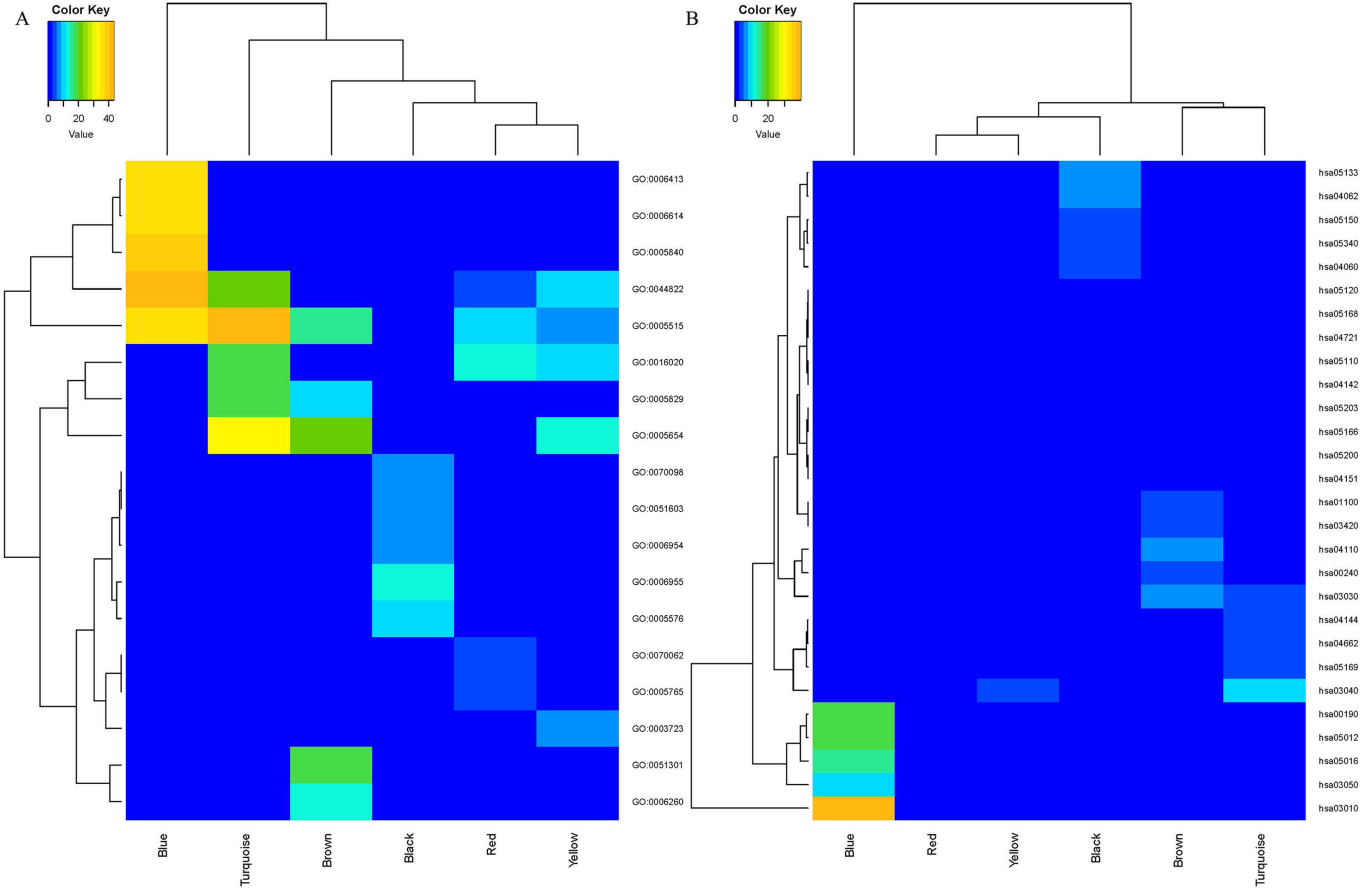
The heatmap for GO (A) and KEGG (B) enrichment analysis of MCL genes in coexpression modules. Rows and columns represent the terms and modules, respectively.

Enrichment analyses of GO and KEGG were conducted to assess the functions of the genes in the six identified modules. The top five enriched GO and KEGG terms with *p* value < 0.05 were selected for further analysis. The heatmap plots for GO (Fig. 3A) and KEGG (Fig. 3B) analysis revealed a large difference in the enriched degree and terms of the coexpression modules. The blue module was determined to be the most important module in enrichment degree after heatmap analysis was conducted (Figs. 3A and 3B). Each module was determined to be significantly different from each other based on analysis of the GO biological process (Table 1). The genes in the black module were primarily enriched in GO:0006955 (immune response), GO:0006954 (inflammatory response), GO:0070098 (chemokine-mediated signaling pathway) and GO:0051603 (proteolysis associated with cellular protein catabolic process). The genes in the blue module were primarily enriched in GO:0006413 (translational initiation) and GO:0006614 (SRP-dependent cotranslational protein aiming at membrane). The genes in the brown module were primarily enriched in GO:0051301 (cell division) and GO:0006260 (DNA replication). The genes in the other three modules were primarily enriched in GO molecular function and cellular components. The results of KEGG pathway analysis are shown in Table 2. The black module was mainly enriched in immune pathways hsa04060: Cytokine–cytokine receptor interaction, and hsa05340: Primary immunodeficiency. The blue module was primarily enriched in the pathways hsa03010: Ribosome, hsa00190: Oxidative phosphorylation (OXPHOS) and hsa03050: Proteasome. The brown module was primarily enriched in cellular processes hsa04110: Cell cycle, hsa03030: DNA replication, hsa00240: Pyrimidine metabolism, hsa01100: Metabolic pathways and hsa03420: Nucleotide excision repair. The red module was enriched in pathways hsa04142: Lysosome and hsa04721: Synaptic vesicle cycle. The turquoise module was principally enriched in Genetic Information Processing hsa03040: Spliceosome and hsa03030: DNA replication. Nineteen genes from the turquoise module were primarily enriched in hsa04662: B cell receptor signaling pathway. The yellow module was primarily enriched in the pathways hsa03040: Spliceosome, hsa05203: Viral carcinogenesis, hsa05166: HTLV-I infection, hsa05200: Pathways in cancer and hsa04151: PI3K–Akt signaling pathway.

**Table 1:** GO enrichment for the genes in the coexpression modules of MCL.

	Term	Count	Percentage	*p* Value
Module black	GO:0006955~immune response	22	18.18181818	2.44E−13
	GO:0005576~extracellular region	36	29.75206612	5.60E−11
	GO:0006954~inflammatory response	17	14.04958678	2.55E−09
	GO:0070098~chemokine-mediated signaling pathway	9	7.438016529	1.71E−08
	GO:0051603~proteolysis involved in cellular protein catabolic process	8	6.611570248	2.22E−08
Module blue	GO:0044822~poly(A) RNA binding	240	14.99063086	3.72E−44
	GO:0005840~ribosome	79	4.93441599	8.92E−41
	GO:0005515~protein binding	963	60.14990631	2.32E−37
	GO:0006413~translational initiation	69	4.309806371	6.67E−37
	GO:0006614~SRP-dependent cotranslational protein targeting to membrane	57	3.560274828	2.80E−36
Module brown	GO:0005654~nucleoplasm	130	34.21052632	5.42E−22
	GO:0051301~cell division	43	11.31578947	1.13E−20
	GO:0005515~protein binding	251	66.05263158	2.41E−16
	GO:0006260~DNA replication	25	6.578947368	1.00E−14
	GO:0005829~cytosol	121	31.84210526	6.12E−12
Module red	GO:0016020~membrane	61	31.28205128	4.66E−13
	GO:0005515~protein binding	137	70.25641026	8.21E−10
	GO:0044822~poly(A) RNA binding	31	15.8974359	4.86E−06
	GO:0070062~extracellular exosome	52	26.66666667	2.53E−05
	GO:0005765~lysosomal membrane	13	6.666666667	2.86E−05
Module turquoise	GO:0005515~protein binding	964	61.51882578	6.97E−44
	GO:0005654~nucleoplasm	389	24.82450542	8.96E−33
	GO:0044822~poly(A) RNA binding	188	11.99744735	6.88E−22
	GO:0016020~membrane	291	18.57051691	4.03E−20
	GO:0005829~cytosol	394	25.14358647	3.53E−19
Module yellow	GO:0005654~nucleoplasm	81	33.75	3.95E−14
	GO:0044822~poly(A) RNA binding	45	18.75	3.59E−11
	GO:0016020~membrane	61	25.41666667	1.62E−09
	GO:0005515~protein binding	156	65	1.01E−08
	GO:0003723~RNA binding	25	10.41666667	1.96E−07

**Note:**

GO, Gene Ontology; SRP, signal recognition particle.

**Table 2:** KEGG pathway enrichment for the genes in the coexpression modules of MCL.

	Term	Count	Percentage	*p* Value
Module black	hsa05133: Pertussis	9	7.438016529	2.87759E−07
	hsa04062: Chemokine signaling pathway	12	9.917355372	6.4214E−07
	hsa04060: Cytokine–cytokine receptor interaction	12	9.917355372	8.78906E−06
	hsa05150: *Staphylococcus aureus* infection	6	4.958677686	0.00011374
	hsa05340: Primary immunodeficiency	5	4.132231405	0.00022661
Module blue	hsa03010: Ribosome	74	4.622111181	2.2086E−40
	hsa00190: Oxidative phosphorylation	49	3.060587133	1.25281E−17
	hsa05012: Parkinson’s disease	50	3.123048095	4.75678E−17
	hsa05016: Huntington’s disease	56	3.497813866	7.26902E−15
	hsa03050: Proteasome	21	1.3116802	3.86976E−10
Module brown	hsa04110: Cell cycle	17	4.473684211	5.56038E−08
	hsa03030: DNA replication	10	2.631578947	1.55272E−07
	hsa00240: Pyrimidine metabolism	13	3.421052632	6.60428E−06
	hsa01100: Metabolic pathways	48	12.63157895	0.000894963
	hsa03420: Nucleotide excision repair	7	1.842105263	0.000996474
Module red	hsa05110: *Vibrio cholerae* infection	5	2.564102564	0.006215548
	hsa04142: Lysosome	7	3.58974359	0.007386343
	hsa04721: Synaptic vesicle cycle	5	2.564102564	0.012146872
	hsa05120: Epithelial cell signaling in *Helicobacter pylori* infection	5	2.564102564	0.014972709
	hsa05168: Herpes simplex infection	8	4.102564103	0.01521917
Module turquoise	hsa03040: Spliceosome	36	2.297383535	1.93556E−09
	hsa05169: Epstein–Barr virus infection	28	1.786853861	5.54839E−06
	hsa04144: Endocytosis	43	2.744097001	1.19036E−05
	hsa04662: B cell receptor signaling pathway	19	1.212507977	1.97499E−5
	hsa03030: DNA replication	13	0.829610721	3.50627E−05
Module yellow	hsa03040: Spliceosome	9	3.75	0.00053903
	hsa05203: Viral carcinogenesis	9	3.75	0.008102343
	hsa05166: HTLV-I infection	10	4.166666667	0.009303826
	hsa05200: Pathways in cancer	12	5	0.021866894
	hsa04151: PI3K–Akt signaling pathway	11	4.583333333	0.022950705

**Note:**

KEGG, Kyoto Encyclopedia of Genes and Genomes; HTLV-I, Human T-cell leukemia virus type 1; PI3K, Phosphatidylinositol-3-kinase; Akt, protein kinase B.

### Hub Gene analysis and identification

**Figure 4:**
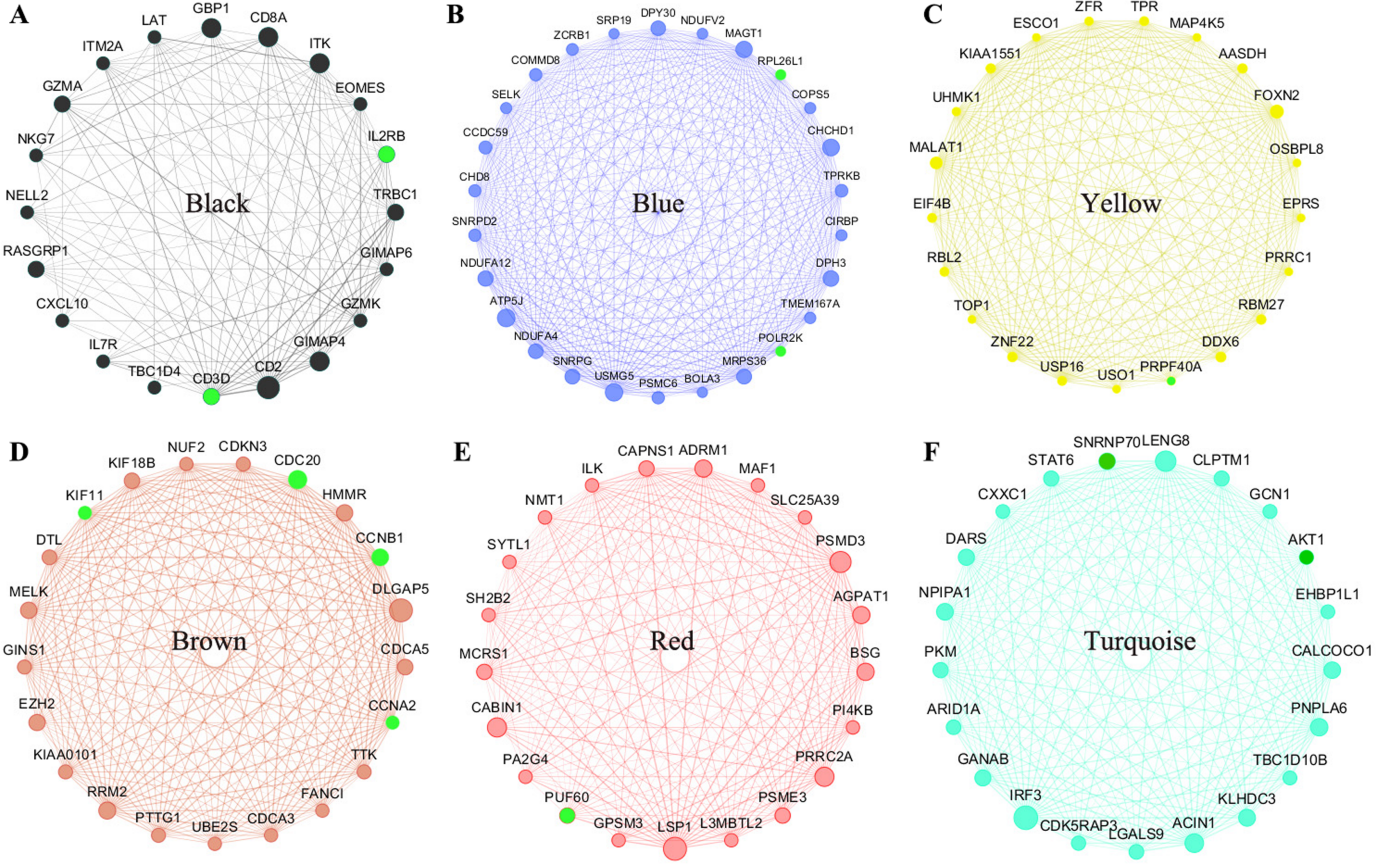
The top genes ranked by weighted degree in each coexpression module (A–F). The green nodes illustrate the real hub genes. Nodes are ordered and sized according to their degree and edges are sized according to their weight.

**Figure 5:**
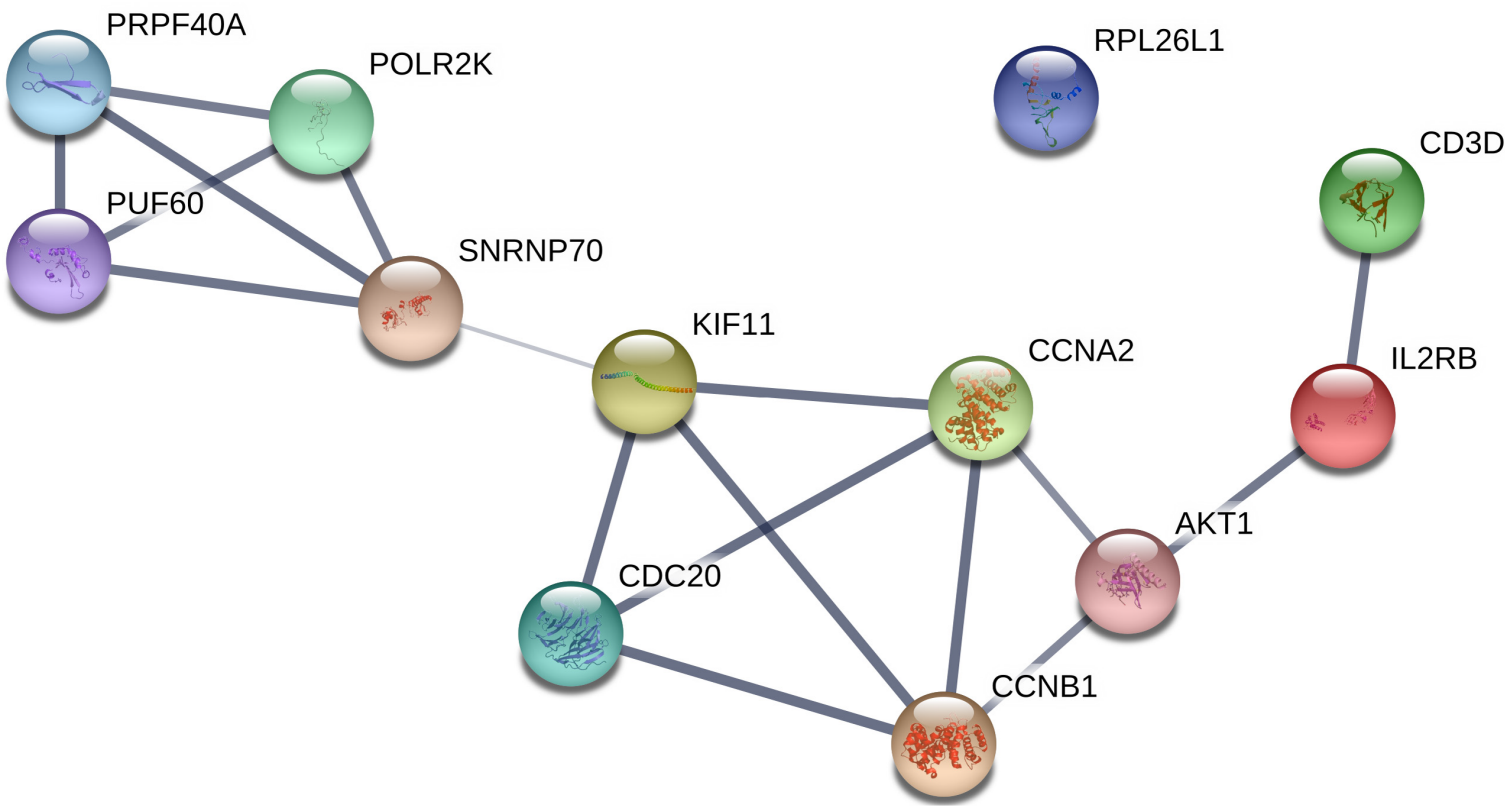
PPI network of the real hub genes.

All of the genes from the six modules were uploaded to the STRING database and a PPI network was constructed using Cytoscape software. The PPI hub genes from the top weighted network were regarded as real hub genes (IL2RB, CD3D, RPL26L1, POLR2K, KIF11, CDC20, CCNB1, CCNA2, PUF60, SNRNP70, AKT1 and PRPF40A) (Fig. 4). The STRING database was used to evaluate the interrelationships of the real hub genes. There was a close correlation among five real hub genes (CDC20, CCNB1, CCNA2, KIF11 and AKT1) (Fig. 5), which indicates their importance.

### Survival analysis

**Figure 6:**
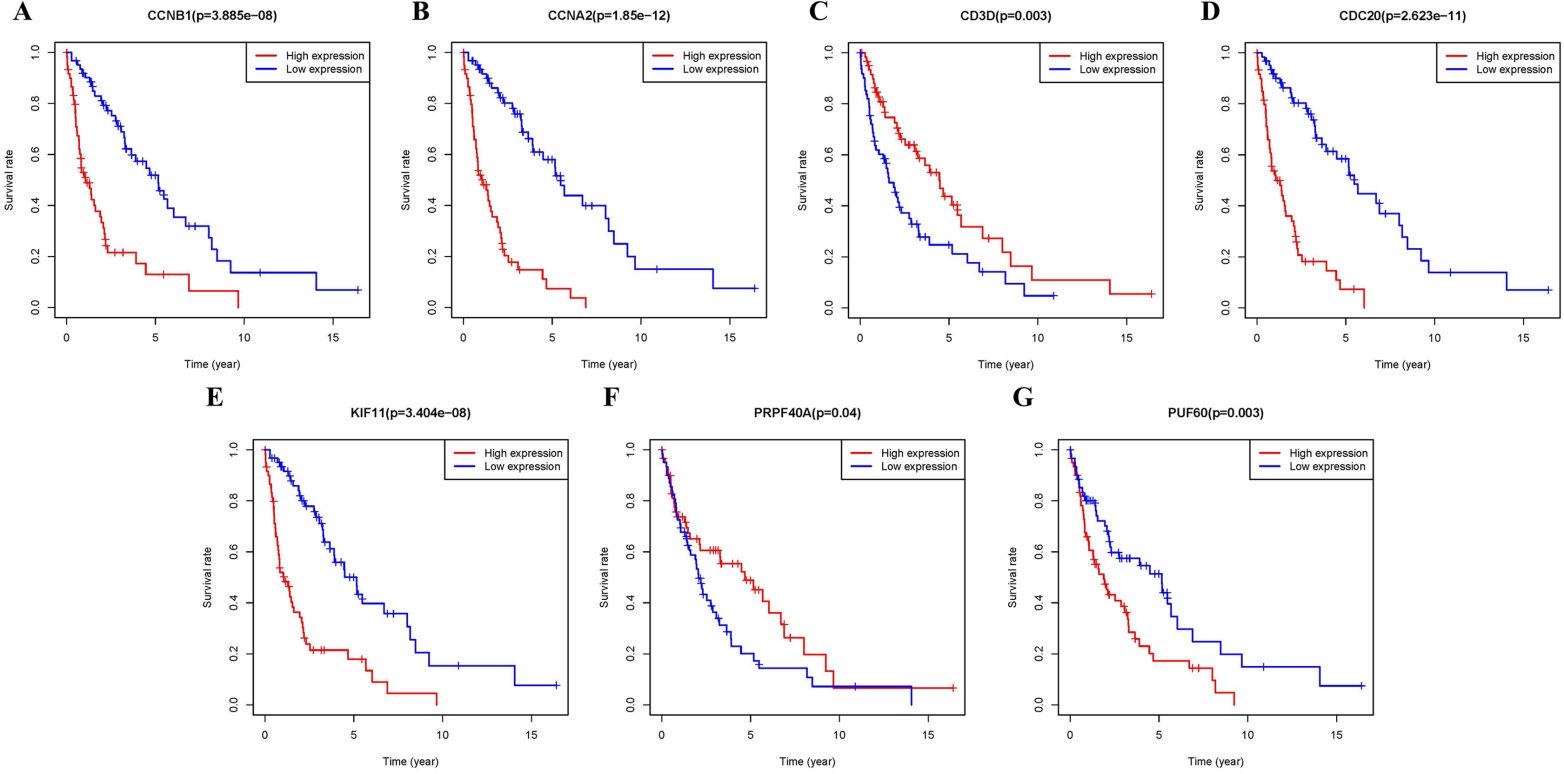
Survival analysis of seven real hub genes expression in MCL patients. Kaplan–Meier survival curves of (A) CCNB1, (B) CCNA2, (C) CD3D, (D) CDC20, (E) KIF11, (F) PRPF40A and (G) PUF60.

Additional survival analysis was conducted on the real hub genes to evaluate their effects on the survival of MCL. Seven genes (KIF11, CDC20, CCNB1, CCNA2, PRPF40A, CD3D and PUF60) (Fig. 6) were found to be associated with overall survival time (*p* < 0.05). Therefore, Kaplan–Meier survival curves indicated that seven genes (KIF11, CDC20, CCNB1, CCNA2, PRPF40A, CD3D and PUF60) may be used as prognostic biomarkers for MCL. Two real hub genes (CD3D and PRPF40A) with high expressions were correlated with longer overall survival which indicated a protective role in MCL biogenesis, while five real hub genes (CCNB1, CCNA2, CDC20, KIF11 and PUF60) were significantly associated with a reduced overall survival rate. Five real hub genes (KIF11, CDC20, CCNB1, CCNA2 and PUF60) were identified as potential prognostic biomarkers for MCL.

### Verification of the real hub genes

**Figure 7:**
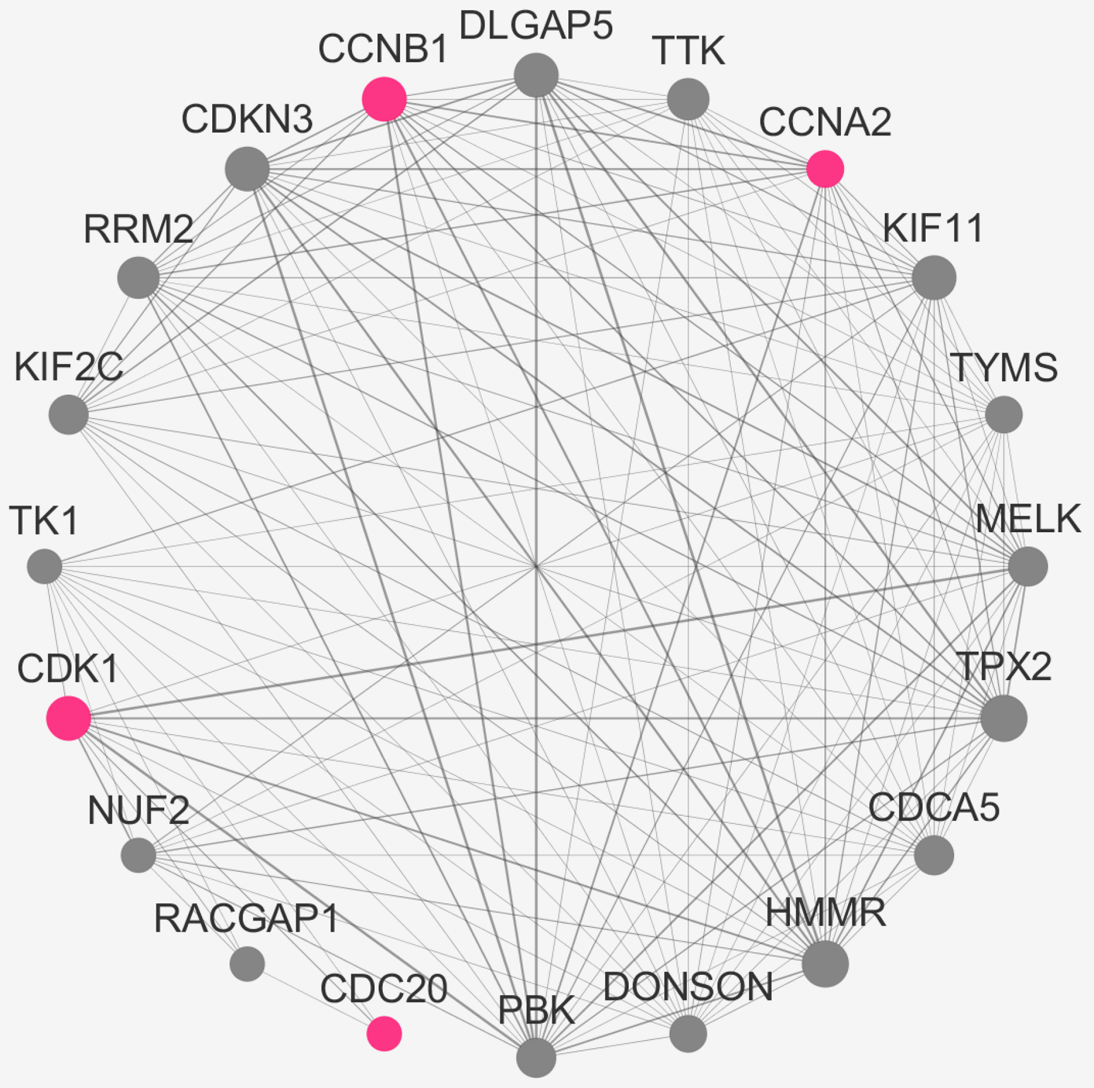
The top genes ranked by weighted degree in the GSE132929. The red nodes illustrate the real hub genes. Nodes are ordered and sized according to their degree and edges are sized according to their weight.

To verify the stability of five real hub genes (KIF11, CDC20, CCNB1, CCNA2 and PUF60), we constructed a coexpression network using WGCNA in the 43 GSE132929 MCL samples. The PPI network was constructed by the STRING database (Fig. S2). The PPI hub genes from the top weighted network were regarded as real hub genes (CDC20, CCNB1, CCNA2 and CDK1) (Fig. 7). We also found that KIF11 appeared in the top weighted network (Fig. 7). Therefore, CDC20, CCNB1 and CCNA2 are stable in GSE132929. And KIF11 is also important in MCL. We failed the validation of PUF60 in GSE132929.

## Discussion

The goal of our research was to establish a gene coexpression network that could be used to forecast the clusters of hub genes involved in the pathogenesis of MCL. WGCNA has a distinct advantage over profiling done by conventional microarray-based expression by targeting a batch of gene modules instead of analyzing genes and their interactions separately. This approach eliminates the potential for error that can occur when taking an independent approach to genes and focuses on molecular transcriptional networks. According to a review of the existing literature, this study is the first to employ WGCNA to detect an array of hub genes as biomarkers related to the pathogenesis of MCL. A total of 123 samples were used to construct the coexpression modules and two samples were excluded as outliers. The tightly co-expressed gene modules with common functional annotations could predict the candidate gene sets underlying a certain biological process and six coexpression modules were identified on this premise. Functional enrichment analysis was carried out in the genes of the modules to identify the critical module. KEGG pathway and GO enrichment analyses were conducted to further investigate the biological functions of the genes enriched among these six modules. The blue module was identified as the most significant module during the diagnosis and progression of MCL. The most critical three KEGG pathways (including Ribosome, OXPHOS and Proteasome) were enriched.

Ribosome, OXPHOS and Proteasome pathways have been found to be closely related to MCL. PF-04691502, a novel phosphoinositide 3-kinase (PI3K)/mTOR inhibitor has potent activity in MCL cell lines, and PF-04691502 decreased phosphorylation of Akt and S6 ribosomal protein. These relationships led Chen et al. (2016) to suggest PF-04691502 as a novel therapeutic agent for MCL patients. Dihydrocelastrol induced apoptosis and cell cycle arrest in MCL cells by inhibiting the (PI3K)/mTOR-mediated phosphorylation of ribosomal protein S6 kinase and eukaryotic initiation factor 4E binding protein (Xie et al., 2018). Inhibition of OXPHOS repressed cell growth in ibrutinib-resistant MCL (Zhang et al., 2019). Targeting metabolic pathways can aid patients with highly refractory MCL. Researchers are in the process of developing an inhibitor of OXPHOS that has already shown efficacy in mouse models of ibrutinib-resistant MCL. The proteasome inhibitor bortezomib was initially approved for the treatment of patients with relapsed MCL (Robak et al., 2015). There is already sufficient evidence for the use of proteasome inhibitors in MCL and these three metabolic pathways can serve as therapeutic targets for patients with MCL.

The real hub genes (including IL2RB, CD3D, RPL26L1, POLR2K, KIF11, CDC20, CCNB1, CCNA2, PUF60, SNRNP70, AKT1 and PRPF40A) were identified by PPI and gene coexpression network analysis. There was a close correlation among five real hub genes (CDC20, CCNB1, CCNA2, KIF11 and AKT1). Survival analysis showed that seven genes (KIF11, CDC20, CCNB1, CCNA2, PRPF40A, CD3D and PUF60) were associated with overall survival. The five hub genes (CDC20, CCNB1, CCNA2, KIF11 and PUF60) were significantly correlated with a shorter overall rate of survival. The results suggest that these five genes could influence the development of MCL treatments. The stability of the hub genes (CDC20, CCNB1 and CCNA2) was confirmed with the same approach in GSE132929. WGCNA is a biology method for interaction and correlation analysis. Therefore, the more data got, the result would be more accurate. The discrepancy in results depends on plenty of reasons such as sample sizes, environmental factors, ethnic origin. Studies with larger sample size are needed in the future.

Emerging evidence reveals that CDC20 plays an important role in tumorigenesis. CDC20 is an oncogene whose overexpression has been observed in numerous cancers (Paul et al., 2017; Sun et al., 2019a) and which plays a pivotal role in mitotic progression. The suppression of the activity of CDC20 can regulate the cell cycle and promote apoptosis (Doğan Şiğva et al., 2019). CDC20 is highly expressed in hepatocellular carcinoma, which is recognized as a predictor of adverse clinical outcomes and an independent prognostic factor (Zhuang, Yang & Meng, 2018). The suppression of CDC20 could mediate the tumor suppressing function of p53 and that CDC20 could be negatively regulated by p53. These findings are consistent with the p53 inactivation observed in various cancer tissues, including acute myeloid leukemia and lung cancer (Hu & Chen, 2015; Simonetti et al., 2019); this effect may be ascribed to CDC20 up-regulation. The anaphase-promoting complex CDC20 can suppress apoptosis by targeting Bim for destruction and ubiquitination. CDC20 has been shown to directly degrade the MLL (KMT2D) protein to assure the progression of the cell cycle during the late M phase. CDC20 fails to interact with MLL fusions in human MLL leukemia cells, which continue to promote the progression of the cell cycle. The deregulated expression of MLL produces the biological change responsible for the constant development of MLL leukemias (Rao & Dou, 2015). Ferrero et al. (2019) recently published the poor prognostic role of MLL (KMT2D) in MCL. The interaction of CDC20 and MLL (KMT2D) in MCL requires further study. CDC20 can predict the overall survival in Diffuse large B-cell lymphoma based on The Cancer Genome Atlas data (Sun et al., 2019a). As a result, the development of specific CDC20 inhibitors may provide a new approach to treat human cancers by virtue of elevated CDC20 expression.

Cancer is characterized by its dysregulation of the cell cycle. CCNB1, the hub gene of the brown module, can promote the transition of cells from the G2 to M phase. However, the overexpression of CCNB1 in cancer results in unchecked cell growth due to its binding to Cdks. The binding of Cdks results in the phosphorylation of other substrates at an improper time and unchecked proliferation (Chiu et al., 2018), which can be ascribed to the inactivation of p53, the tumor suppressor protein. The wild-type p53 has been reported to suppress the expression of cyclin B1 (Lu et al., 2016). High CCNB1 expression is seen in a diverse number of cancers, including esophageal, gastric and colorectal cancers (Ding et al., 2018; Wen et al., 2017). The high expression level of cyclin B1 is also correlated to the degree of tumor invasion and metastasis (Gu et al., 2019). The down-regulation of cyclin B1 results in tumor regression, making CCNB1 an attractive target for further study.

CCNA2 is a hub gene found in the brown module. It can promote the transition of G1/S and G2/M phases in the cell cycle. The up-regulated CCNA2 expression is found in numerous types of cancer, including pancreatic ductal adenocarcinoma and colorectal cancers (Dong et al., 2019; Gan et al., 2018). An increased CCNA2 expression can promote tumorigenesis. Recent experimental data indicates that CCNA2 is a potential target for cancer therapy. The kinesin family member 11(KIF11) is one of the kinesin family motor protein members and is associated with spindle formation and tumor genesis (Jin et al., 2019). High KIF11 expression contributes to tumor progression and is associated with unfavorable prognosis (Jin et al., 2019; Pei et al., 2017; Piao et al., 2017). KIF11 inhibitors have been developed as chemotherapeutic agents to treat cancer. Poly-U binding splicing factor 60 kDa (PUF60), a factor regulating RNA splicing, is closely involved in tumor progression. The expression of PUF60 was highly correlated with TNM staging and lymph node metastasis in breast cancer (Sun et al., 2019b). Kobayashi et al. (2016) detected PUF60 auto-antibodies in the sera of early-stage colon cancer patients and concluded that it may be a candidate biomarker for the diagnosis and prognosis of colon cancer.

## Conclusion

Significant key pathways and the hub genes related to the prognostic biomarkers of MCL were identified using comprehensive analysis and a bioinformatic approach. The development of the potential and targeted selective inhibitors for key pathways and the extractive real hub genes, including CDC20, CCNB1, CCNA2, KIF11 and PUF60, may provide a novel treatment opportunity for MCL therapy. However, these significant pathways and hub genes should be analyzed and validated in future clinical studies to determine the biological targets that are most effective for MCL.

## Supplemental Information

10.7717/peerj.8843/supp-1 Supplemental Information 1 The influence of different soft threshold power on the average connectivity degree of coexpression modules of MCL genes.

10.7717/peerj.8843/supp-2 Supplemental Information 2 PPI network in the GSE132929.

## Additional Information and Declarations

### Data Availability

The following information was supplied regarding data availability: Data is available at NCBI GEO: GSE93291.
