# Supplementary figures and images for: Identification of key gene modules and hub genes of human mantle cell lymphoma by coexpression network analysis
Source: PeerJ. 2020 Mar 20;8:e8843. doi: 10.7717/peerj.8843 (PMC7087492; doi:10.7717/peerj.8843)

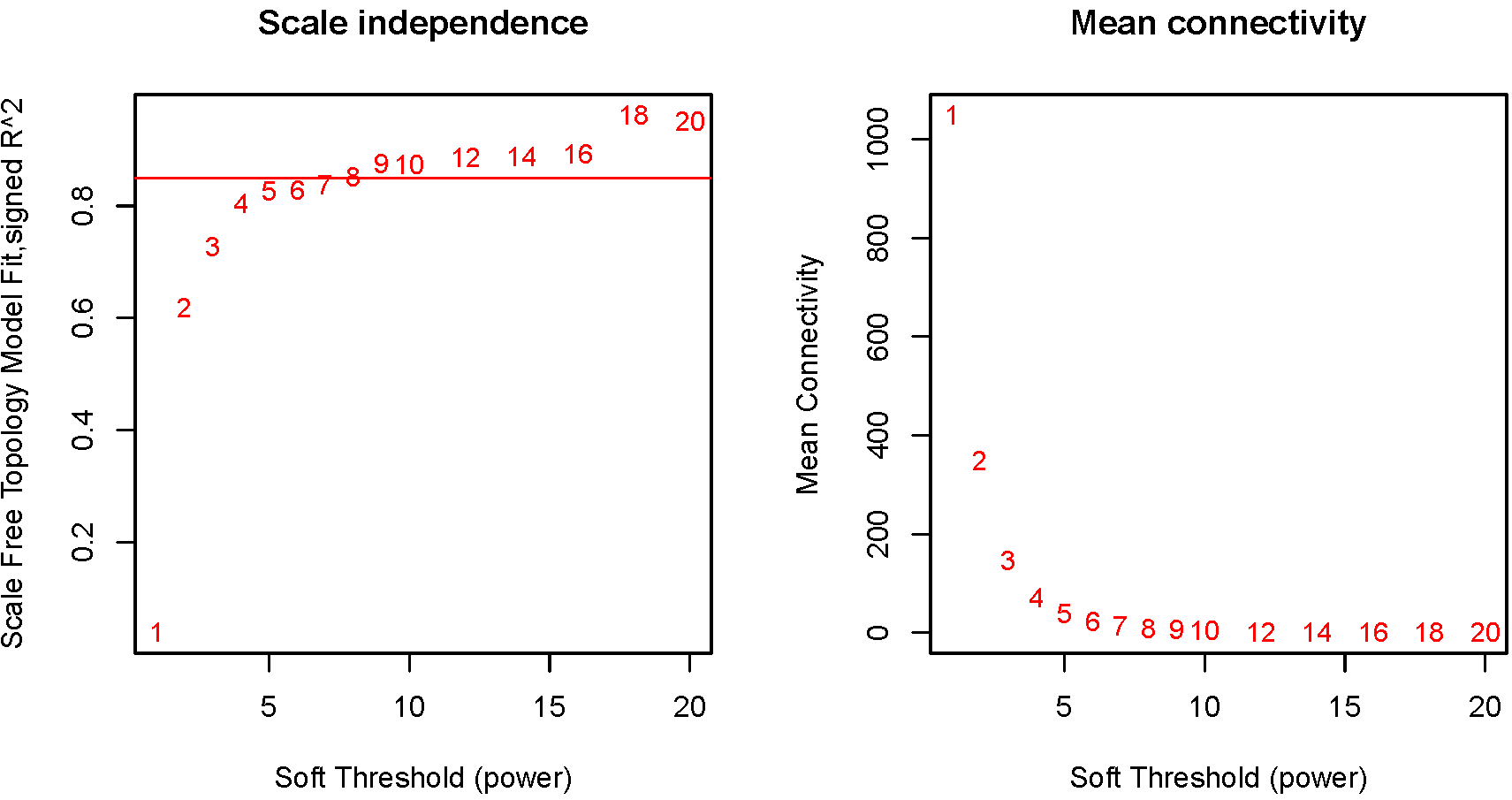

Supplement: Supplemental Information 1 [file peerj-08-8843-s001.png]

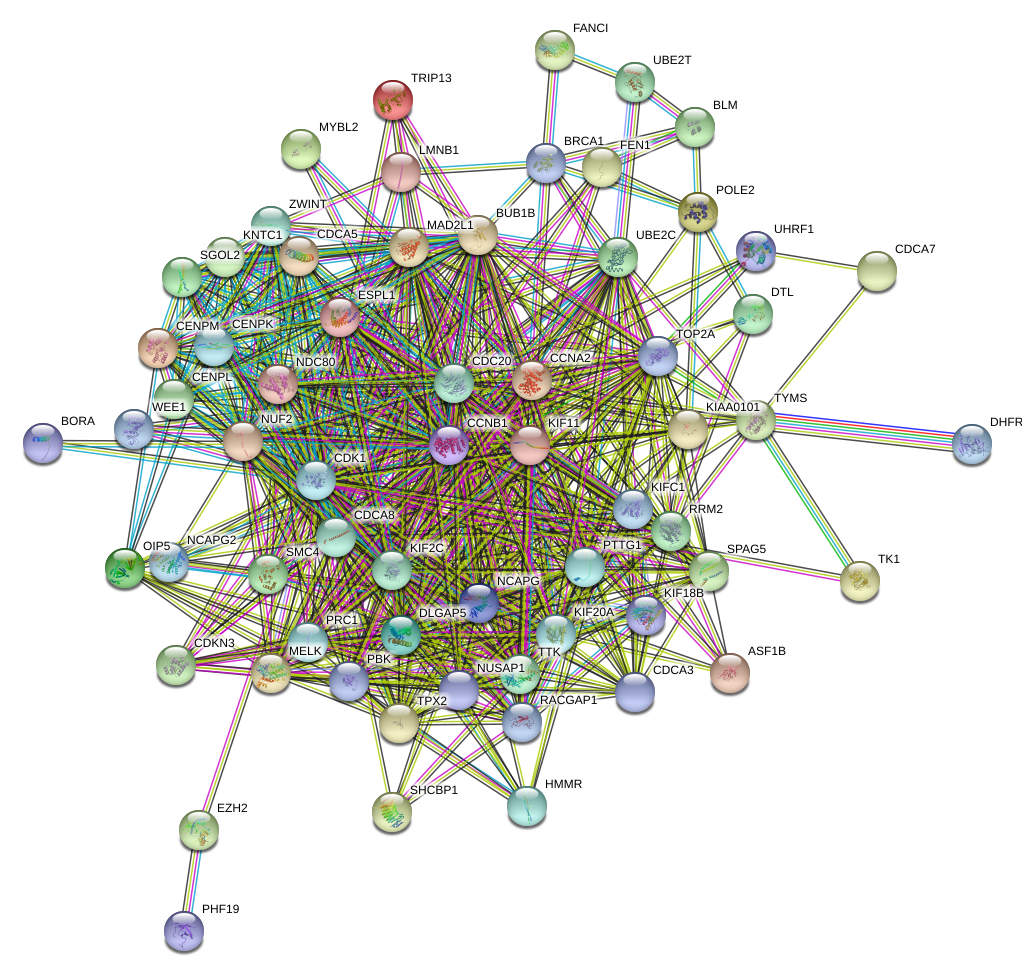

Supplement: Supplemental Information 2 [file peerj-08-8843-s002.png]
